# Labor in Mature Primiparæ

**Published:** 1898-04

**Authors:** 


### LABOR IN MATURE PRIMIPARÆ.

DeKoninck (*Revue Med.; Brit. Med. Jour.*) has compiled an instructive memoir on labor in primiparæ married for some years and relatively mature (twenty-eight years Bidd and Pourtad, thirty-two Ahlfeld). DeKoninck gives thirty as the earliest year coming under "maturity," the "primipares agees" of the French authorities. He sets aside as curiosities certain cases of primiparæ almost "aged" in the English sense of the term, such as Cohnstein's two women aged fifty, and Steinmann's woman aged fifty-two. It appears that in a genuine uncomplicated case of delayed impregnation the advent of the catamenia is always found to have occurred late in youth.

Out of four hundred and one such cases menstruation was retarded till twenty in thirty-nine, till twenty-four in four, and till twenty-six in one. As to the retarded first pregnancy, abortion, ectopic gestation, twins and special renal mischief are relatively frequent. Above all, lingering labor is specially common, statistics even exceeding guesses and *a priori* reasoning in this respect. In thirteen out of seventeen noted by DeKoninck labor lasted from forty to fifty hours, the remaining labors being yet longer; one exceeded ninety hours.

Feebleness of uterine contraction is absolutely from first to last, and independent of any obstetrical combination. They also cause far more physical and mental exhaustion than the vigorous contractions of a young uterus, and at the same time are more painful. There are other discrepancies in the "pains" seen in mature primiparæ of the same age, probably homologous with the great variations in the age of the menopause observed in otherwise normal women. The uterus may be older in one woman aged thirty-five than in another of the same age.

The forceps and other obstetrical operations are often required in the mature. Most of the above facts are easily explained. The excess of male infants borne by mature primiparæ (thirty per cent.) is a less explicable phenomena. Hetker considers the predominance of male infants as a specialty of all primiparæ, but Rumpe turns attention to the fact that in a family of children the predominance of males is commoner the further the mother is from her first menstrual period.

